# Development of an indirect ELISA using a novel linear epitope at the C-terminal region of the VP2 protein to specifically detect antibodies against Senecavirus A

**DOI:** 10.1186/s12985-022-01934-8

**Published:** 2022-12-02

**Authors:** Zhongyuan Ma, Jianliang Lv, Zhongwang Zhang, Li Pan

**Affiliations:** State Key Laboratory of Veterinary Etiological Biology, National Foot-and-Mouth Disease Reference Laboratory, Chinese Academy of Agricultural Sciences, Lanzhou Veterinary Research Institute, Lanzhou, Gansu China

**Keywords:** Diagnosis, Senecavirus A, VP2, Epitope, ELISA

## Abstract

**Background:**

Senecavirus A (SVA) is a pathogen that has recently caused porcine idiopathic vesicular disease (PIVD). The clinical signs are similar to those of foot-and-mouth disease, porcine vesicular disease, and vesicular stomatitis. Therefore, identification of SVA as a cause of PIVD is important to eliminate this emerging pathogen.

**Methods:**

In this study, an indirect ELISA based on the VP2 epitope (VP2-epitp-ELISA) was developed to detect antibodies directed against SVA.

**Results:**

A novel linear epitope (^271^GLRNRFTTGTDEEQ^284^) was first identified at the C-terminus of the VP2 protein by epitope mapping. The diagnostic performance of VP2-epitp-ELISA was estimated by testing a panel of known background sera from swine. Under the optimum test conditions, when the cutoff value was 37%, the diagnostic sensitivity (Dn) and diagnostic specificity (Dp) of the assay were 91.13% and 91.17%, respectively. The accuracy of VP2-epitp-ELISA was validated and further compared with that of commercial diagnostic kits. The diagnostic results showed that VP2-epitp-ELISA did not cross-react with serum positive for other idiopathic vesicular diseases and had a concordance rate of 90.41% with the Swinecheck^®^ SVA bELISA.

**Conclusions:**

These results indicate that VP2-epitp-ELISA is suitable for specific detection of antibodies against SVA in swine.

## Background

Senecavirus A (SVA)—also known as Seneca Valley virus (SVV)—was isolated from contaminated PER. C6 cells in 2002 [1]. SVA is a nonenveloped, positive-sense, single-stranded RNA virus. SVA belongs to the family *Picornaviridae*, genus *Senecavirus*. The first SVA genome determined in 2007 (SVA-001 strain) has typical genomic features of other picornaviruses, including the standard L-4-3-4 layout [2, 3]. The genome of SVA contains a single open reading frame (ORF) encoding four structural proteins (SPs: VP4, VP2, VP3 and VP1) and eight nonstructural proteins (NSPs: L, 2A, 2B, 2C, 3A, 3B, 3C and 3D), and the four structural proteins constitute icosahedral viral particles with a diameter of ∼30 nm [4].

SVA infection was first reported to be associated with porcine idiopathic vesicular disease (PIVD) in Canada. Serological surveys have revealed that SVA has spread widely in the USA, Brazil and China [5–7]. According to recent studies, the clinical signs induced by SVA infection are indistinguishable from those of foot-and-mouth disease, porcine vesicular disease, and vesicular stomatitis [8, 9]. Therefore, identification of SVA as a cause of PIVD is important to eliminate this emerging pathogen.

SVA has only one serotype, and swine are thought to be a natural host of SVA. SVA infection develops robust neutralizing antibody responses (NAs) in herds regardless of the clinical manifestations of the disease [10, 11]. In the early stage of the disease, NAs are predominantly composed of IgM antibodies, and SVA-specific IgG antibodies appear later and are detected in the serum on day 7 post-infection [12, 13]. Importantly, VP1 and VP3 IgG antibodies are undetectable following resolution of the disease, while VP2 IgG antibodies can persist for up to 35 days after SVA infection [14]. Thus, VP2 is an ideal diagnostic target for specific detection of antibodies against SVA.

At present, three diagnostic methods are used to detect antibodies against SVA. One method is based on virus neutralization tests (VNT), and the other two methods are blocking enzyme-linked immunosorbent assay (bELISA) using monoclonal antibodies and indirect ELISA (iELISA) using VP2 protein [14–16]. However, diagnostic methods based on epitopes to detect antibodies directed against SVA are lacking.

In this study, a novel linear epitope (^271^GLRNRFTTGTDEEQ^284^) that recognizes SVA-infected sera was first identified at the C-terminus of VP2 by epitope overlap mapping. Meanwhile, an indirect ELISA based on the VP2 epitope (VP2-epitp-ELISA) was also developed for specific detection of antibodies against SVA, and then, the diagnostic performance of VP2-epitp-ELISA was estimated by testing a panel of known-background sera from swine.

## Results

### Viral antigen production

**Fig. 1:**
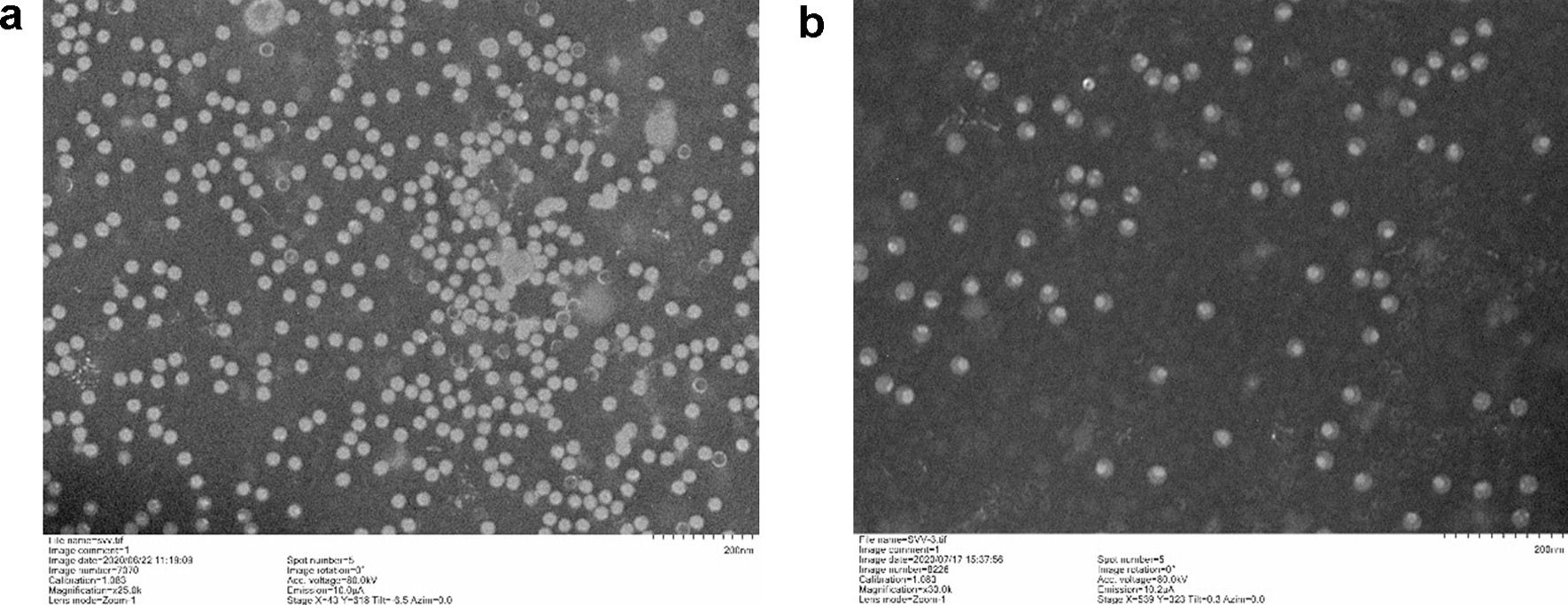
Viral antigen production and purification. a and b. Virus particles and BEI-inactivated viruses were purified by ultrahigh-speed centrifugation and sucrose gradient centrifugation respectively, and purified viruses were then used for preparation of infected and immune sera. As shown in Fig. 1**a** and **b**, the diameter of the virus particles was approximately 30 nm (bar = 200 nm)

To obtain large batches of viruses, NCI-H1299 cells were infected with SVA. When the CPE occurred, the virus solution was collected after the cells were frozen and thawed three times. Viral bands were obtained by ultrahigh-speed centrifugation and sucrose gradient centrifugation, and the purified virus was confirmed by TEM. As shown in Fig. 1a, the diameter of the virus particle is approximately 30 nm. For SVA inactivation, the clarified viral supernatant was incubated with 10 mM binary ethylenimine (BEI) for 24hr at 37 °C. The reaction was terminated by the addition of 10% sodium thiosulfate, and the inactivated virus is shown in Fig. 1b, the structure of the SVA genomes were interrupted by BEI.

**Fig. 2:**
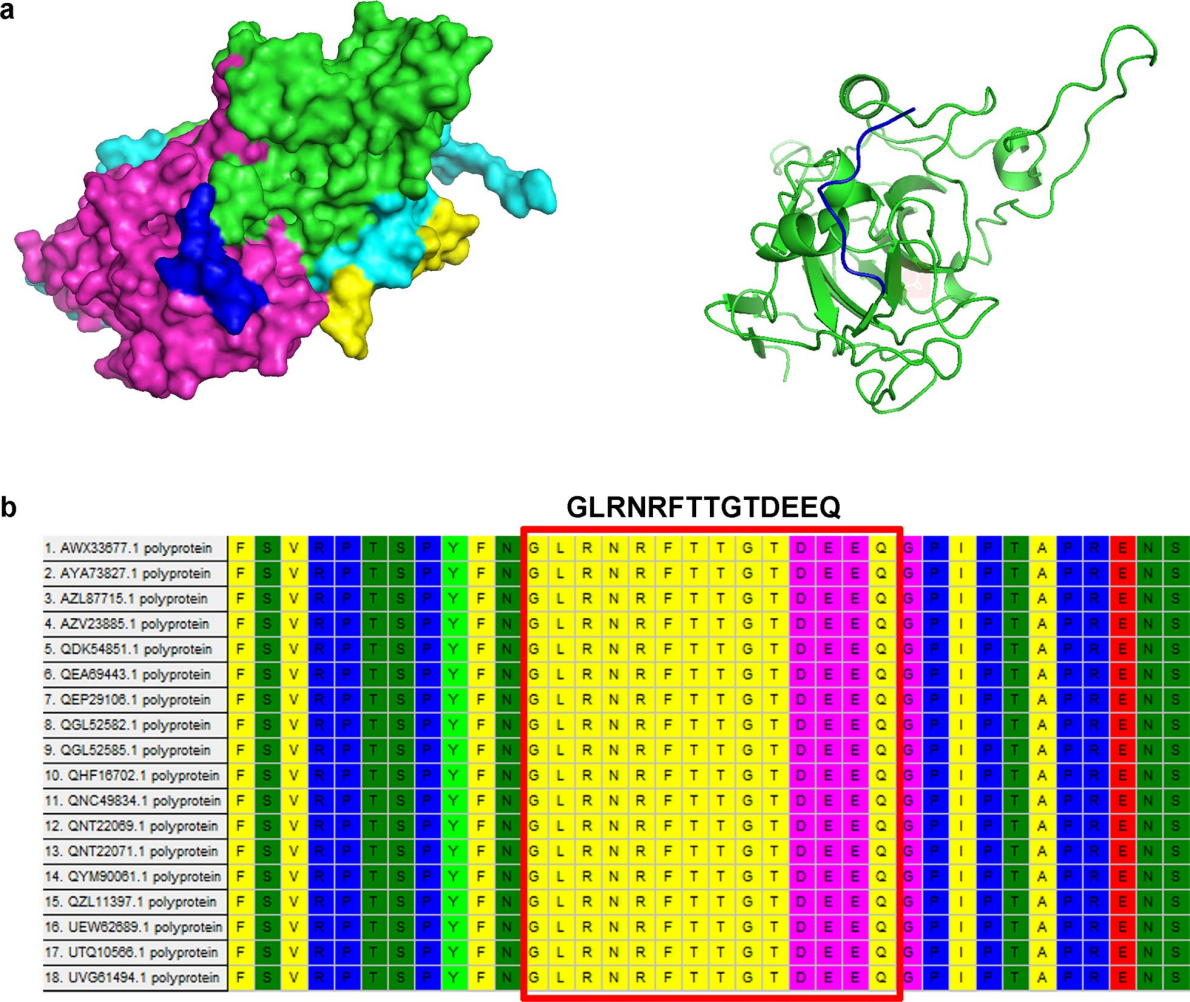
Character analysis of the linear B-cell epitope of the VP2 protein. **a** The relative spatial position of the identified epitope is presented in a surface view from a partially predicted 3D structure of SVA VP2 (the reference structure, PDB ID code 3CJI). The epitope recognized by SVA-infected serum is shown in blue. **b** Amino acid alignment was performed on different SVA isolates and VP2 proteins using MEGA software. The linear epitope ^271^GLRNRFTTGTDEEQ^284^ is shown inside the red box

### Identification of VP2 major linear B-cell epitopes

To define major epitopes on the SVA VP2 protein, peptides representing the whole polypeptide of SVA-VP2 were synthesized and then analyzed by indirect ELISA. Notably, only the C-terminal peptide of VP2 (^271^GLRNRFTTGTDEEQ^284^) can recognize different SVA-infected serum samples. To understand the structural mechanism of the epitope identified in SVA-infected serum, the X-ray crystal structure of SVV-001 (PDB ID code 3CJI) was used as the reference structure, and the peptide was analyzed using an online computer software program (PyMOL2.5). The results revealed that the C-terminal epitope is fully exposed on the surface of the predicted SVA. Sequence alignment analysis of this epitope from the different SVA isolates was performed to determine if the identified epitopes were conserved among different SVA reference strains. The results showed that the epitope ^271^GLRNRFTTGTDEEQ^284^ was conserved in SVA VP2. (Fig. 2)

### Standardization of VP2-epitp-ELISA

**Fig. 3:**
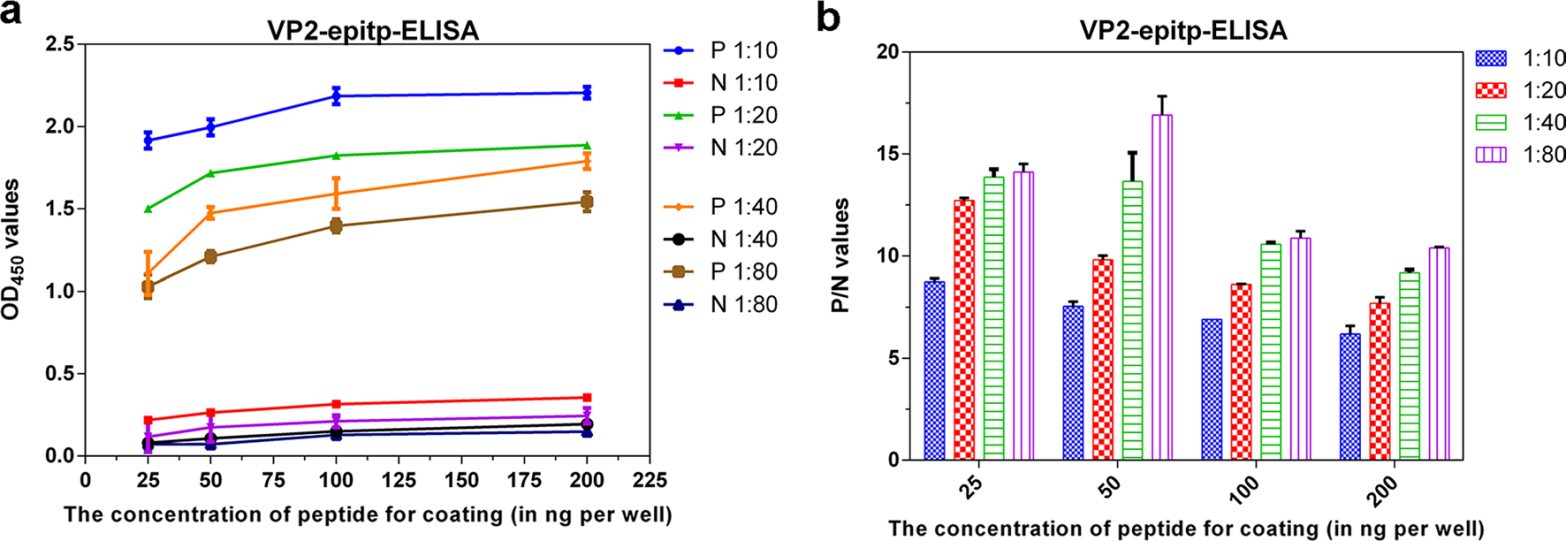
Results of checkerboard titration assays used to optimize peptide concentrations and serum dilution. **a** Standard positive (*P*) and standard negative (*N*) control serum samples were serially diluted 2-fold (1:10, 1:20, 1:40, 1:80), and the coating concentration of the identified peptide was varied from 25ng/well to 200ng/well. **b** When the conditions were a coating concentration of the peptide of 50 ng/well and a standard control serum sample (*P* and *N*) dilution ratio of 1:80, and the best signal-to-noise ratio (*P*/*N* value) of VP2-epitp-ELISA was 17

The optimum peptide coating concentration and the serum dilution were determined using a checkerboard titration method (Fig. 3). The concentration of the peptide and the test serum dilution were fixed at 50 ng/well and 1:80, respectively. Under the optimal conditions, the OD_450_ value of standard positive control sera was 1.232, the OD_450_ value of standard negative control sera was 0.072, and the signal-to-noise ratio reached (P/N) 17.

### Determination of the cut‑off value, Dn, and Dp for VP2-epitp-ELISA

**Fig. 4:**
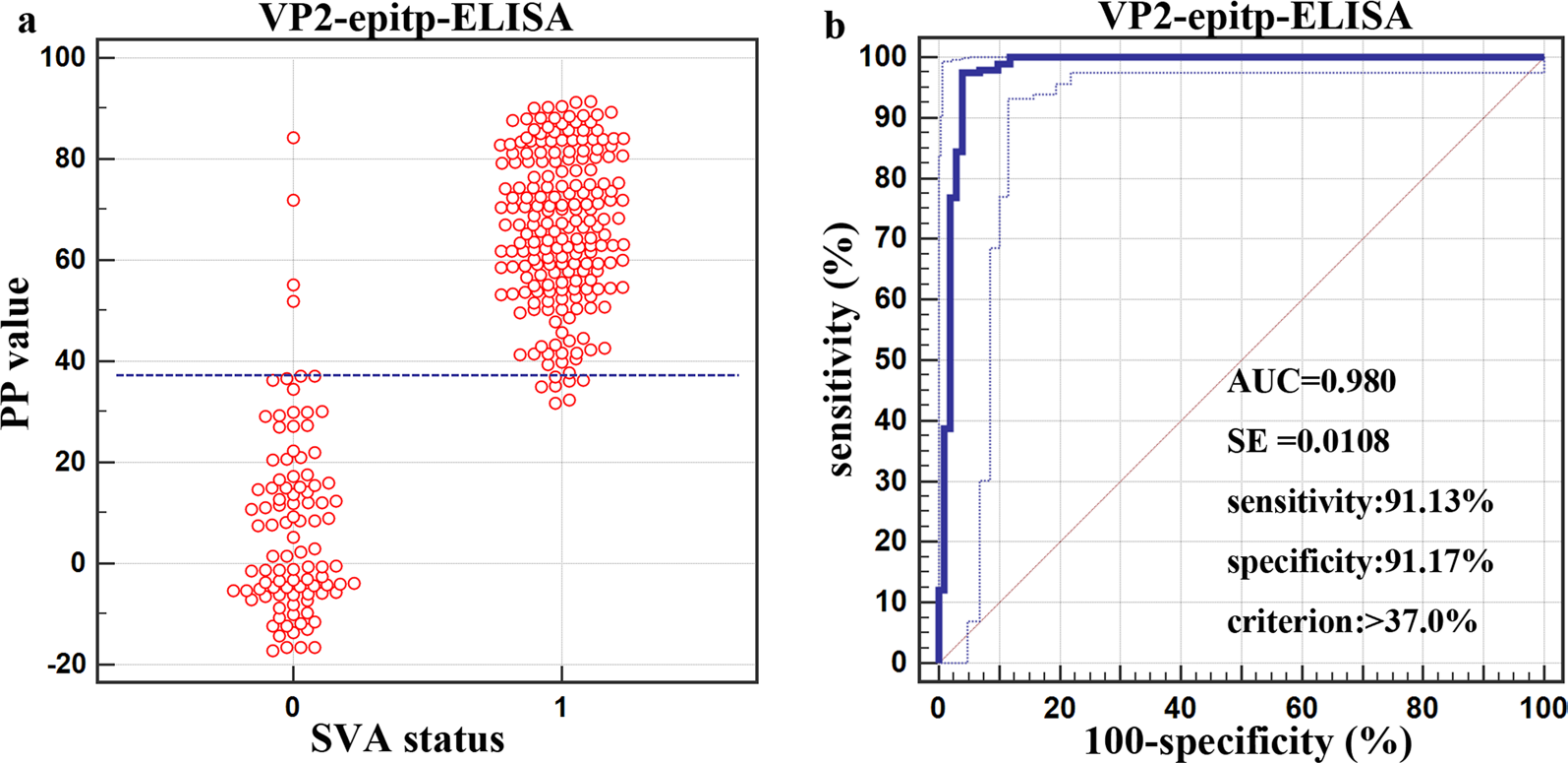
Estimation and comparison of the Dn and Dp values of the assay by analyzing the receiver operating characteristic (ROC) curve. **a** Interactive dot diagram of the VP2-epitp-ELISA in testing sera from swine: 0, negative serum samples (*n* = 102) from clinically healthy and unvaccinated animals; 1, positive serum samples (*n* = 124) from vaccinated animals. Each point on the dot diagram represents a serum sample with a known statue, and associated with a particular PP value. **b** Each point on the ROC plot represents a sensitivity-specificity pair associated with a particular threshold. The best Dn and Dp values were 91.13% and 91.17%, respectively, and the criterion was 37%

The PP values of the 226 serum samples (serum samples from naïve animals, negative, *n* = 102; serum samples from vaccinated animals, positive, *n* = 124) were used to estimate the Dp and Dn by analyzing the receiver operating characteristic (ROC) curve (Fig. 4). When the cutoff PP value of VP2-epitp-ELISA was 37%, the Dn and Dp of cCLIA were 91.36% and 92.41%, respectively. The area under the ROC curve (AUC) was 0.980 (standard error, SE = 0.0108), with a 95% confidence interval (CI) of 0.957 to 0.993.

### Accuracy rates and diagnostic performance

The samples mentioned above (*n* = 226) were also examined using a commercial diagnostic kit to evaluate and compare the accuracy of the test methods (Table 1). The accuracy rate of the VP2-epitp-ELISA (91.17%, 91.12%) was nearly equivalent to that of the Swinecheck^®^ SVA bELISA (96.07%, 95.16%). The concordance rate between the VP2-epitp-ELISA and Swinecheck^®^ SVA bELISA was 90.41% (151 of 167) across field serum samples.

**Table 1:** Comparison of the diagnostic performances of the VP2-epitp-ELISA and Swinecheck^®^ SVA bELISA

Sample details	Sample size	VP2-epitp-ELISA		Swinecheck^®^ SVA bELISA	
		Positive	Negative	Positive	Negative
Naïve serum	102	9	93	4	98
Accuracy rate		91.17%		96.07%	
Vaccinated serum	124	113	11	118	6
Accuracy rate		91.12%		95.16%	
Field serum	167	90/167	77/167	99 (88)/167	68 (63)/167
Concordance rate		90.41% (151/167)			

## Discussion

In recent years, the SVA has spread rapidly and has caused economic losses to the pig industry [8, 17]. Sero-monitoring has revealed that pigs are often coinfected with SVA and other vesicular viruses, and the clinical symptoms of SVA and other vesicular viruses are difficult to distinguish [18]. As no SVA commercial vaccines have been developed, immunization with the FMDV vaccine shows no cross protection against SVA infection [19]. Therefore, the specific diagnosis of SVA infection is crucial to its prevention and control.

Previous studies have shown that antibody responses to VP2 were higher than those to VP1 and VP3, and VP2 would be a reliable target to detect antibodies directed against SVA [14]. The structural protein of SVA is composed of four structural proteins, VP4, VP2, VP3, and VP1, which contain the major epitope region [20, 21]. To screen specific linear epitopes, we prepared high-purity SVA particles for preparation of infected and immune sera. Simultaneously, peptides representing the whole polypeptide of SVA-VP2 were synthesized and a conserved linear epitope (^271^GLRNRFTTGTDEEQ^284^) was identified at the C-terminal region of VP2 by epitope mapping. To verify the reliability of this epitope as a diagnostic antigen, a panel of serum samples was tested by using VP2-epitp-ELISA and Swinecheck^®^ SVA bELISA, and the test results of the two assays were analyzed using MedCalc software and compared with the status of the serum samples.

Notably, the structural analysis showed that ^271^GLRNRFTTGTDEEQ^284^ is fully exposed on the surface of SVA particles, and the results showed that this epitope can recognize different SVA-infected serum samples. In addition, the test results showed that this epitope did not cross-react with sera positive for other idiopathic vesicular diseases.

## Conclusions

In this study, an indirect ELISA based on the VP2 epitope was developed for specific detection of antibodies against SVA. Based on the results of the study, the newly developed VP2-epitp-ELISA had high discriminatory power, diagnostic sensitivity (91.13%) and diagnostic specificity (91.17%). Therefore, VP2-epitp-ELISA is a promising tool for detecting antibodies against SVA in large-scale serological surveys. Additionally, the epitope recognized by SVA-infected sera may provide insights for novel SVA diagnostic approach development.

## Methods

### Serum samples

Serum samples from naïve animals: Serum samples from clinically healthy and unvaccinated pigs (n = 102) were collected and tested using Swinecheck^®^ SVA bELISA kits (all samples had negative results, blocking rate < 50%). These samples were used to assess diagnostic specificity (Dp) and the cutoff value.

Serum samples from vaccinated animals: A total of 124 serum samples were collected at 28–42 days post-vaccination (dpv) with an inactivated whole-virus vaccine and collected by our research group. These samples were used to assess diagnostic sensitivity (Dn) and the cutoff value.

Serum samples from infected animals: A total of 23 serum samples from swine infected with SVA ZJ/2015 at 7–14 days post-infection (dpi) were collected by our research group. These serum samples were used for epitope mapping after heat inactivation.

Field samples (partial samples seropositive for other vesicular diseases): A total of 167 serum samples from swine were collected in the field and preserved at -80°C. These serum samples were used to compare accuracy rates and diagnostic performances.

Standard control sera: SVA-positive sera (blocking rate 101.2%, measured by Swinecheck^®^ SVA bELISA kit and confirmed by VNT) were collected from the vaccinated group, and SVA-negative sera (blocking rate 9.2%, measured by Swinecheck^®^ SVA bELISA kit and confirmed by VNT) were collected from the naïve group. A standard positive control (P) and negative control (N) were created as internal controls.

### Growth and purification of SVA

NCI-H1299 cells (ATCC) were grown in Dulbecco’s modified Eagle’s medium (DMEM; Gibco, Waltham, MA, USA) supplemented with 10% fetal bovine serum (FBS) and 1% penicillin‒streptomycin. The SVA ZJ-2015 strain was preserved in our laboratory. Briefly, NCI-H1299 cells infected with SVA ZJ-2015 were collected and subjected to three freeze‒thaw cycles. Cellular debris was removed by centrifugation at 6000 × g for 30 min at 4 °C. The virus was then precipitated with 8% polyethylene glycol (PEG 8000) and 0.5 M NaCl for 16 h at 4 °C. The precipitated virus was further centrifuged by a 10–50% sucrose density gradient. Subsequently, the virus band was taken up for desucrose treatment, and the precipitate was resuspended in PBS (pH = 7.4) and stored at − 80°C. Purified virus was used for the preparation of infectious and immune sera.

### Neutralization assay

The neutralizing activity of sera was determined by end-point dilution assay. The test sera were heat-inactivated at 56 °C for 30 min and then twofold serially diluted in DMEM (50 µl/well). Each dilution was repeated in triplicate. An equal volume of 100 median tissue culture infective dose (TCID50) SVA ZJ-2015 was added to each well of a 96-well tissue culture microtiter plate (50 µl/well). The plates were incubated for 1 hr at 37 °C. Then, 100 µl of NCI-H1299 cells (2×10^4^ cells) in DMEM were added to each well, and the plates were incubated at 37°C in a 5% CO2 incubator. The cytopathic effect (CPE) was scored after 72 hr. The neutralizing antibody titer was calculated by the Reed–Muench method. In brief, a 100% CPE on cells in the tested serum wells was judged as negative, and the presence of more than 50% of cells remaining was judged as positive. Finally, the serum dilution that protected 50% of the cell wells from cytopathy was calculated, and this dilution was the serum neutralizing antibody titer.

### Epitope mapping of VP2 protein

To identify the epitope recognized by SVA-infected pig serum, overlapping peptides of the VP2 protein (15 amino acids in length, overlapping each other by 10 amino acids) were synthesized by GenScript Biotech Corporation (Nanjing, China). The synthesized peptides were then screened and identified by indirect ELISA.

### Construction and optimization of VP2-epitp-ELISA

ELISA plates (Costar, catalog number: 42592) were coated at 4 °C overnight with 100 µl of synthesized peptides (200 ng/well, 100 ng/well, 50 ng/well, 25 ng/well) diluted in PBS buffer (pH 7.4). The plate was then thoroughly washed with PBST (PBS containing 0.05% Tween-20) and blocked with PBST containing 1% BSA and 5% sucrose at 37 °C for 2 h. After five PBST washes, positive and negative control serum samples were diluted (1:10, 1:20, 1:40, 1:80) in the abovementioned sample diluent, diluted sera were transferred to coated plates at 100 µl per well, and the plate was incubated at 37 °C for 30 min. Then, after five washes, each well received 100 µl of 1:10000 diluted secondary antibodies and rabbit anti-pig IgG antibodies conjugated to horseradish peroxidase (Sigma, USA) for 30 min at 37 °C. After washing, the plate was developed with 100 µl of TMB substrate at 37 °C for 10 min, and the reaction was terminated with 100 µl of 2 M H_2_SO_4_. The absorbance at 450 nm (A_450_) was measured using a Varioskan Lux instrument.

### Estimation of the cutoff value, Dn and Dp for VP2-epitp-ELISA

After the optimum coating concentration and serum dilution were confirmed, pig serum samples with a known status were tested using VP2-epitp-ELISA to evaluate their Dp, Dn and cutoff value. The A_450_ values of the positive control (A_450_ pos) and test samples (A_450_ sample) were corrected by deducting the A_450_ value of the negative control (A_450_ neg). The sample results were recorded as percent positive (PP) using the following formula: PP = (A_450_ sample – A_450_ neg) ×100%/ (A_450_ pos – A_450_ neg). The A_450_ values were also recorded as the percent inhibition (PI) using the same formula. All PP values of the assay were used to estimate the cutoff value, Dp and Dn using MedCalc software.

### Comparison of accuracy rates and diagnostic performances

To evaluate the accuracy rates and concordance rate of VP2-epitp-ELISA, a total of 249 serum samples (serum samples from naïve animals: 102; serum samples from vaccinated animals: 124) and a total of 167 field samples (partial samples seropositive for other vesicular diseases) were tested using Swinecheck^®^ SVA bELISA.

## Data Availability

All data generated or analyzed in this study are included in this published article.

## Abbreviations

SVA: Senecavirus A
PIVD: Porcine idiopathic vesicular disease
ORF: Open reading frame
NAs: Neutralizing antibody responses
ELISA: Enzyme-linked immunosorbent assay
CPE: Cytopathic effect
